# Supplementary material for: Synthesis, Antitumor Evaluation, Molecular Modeling and Quantitative Structure–Activity Relationship (QSAR) of Novel 2-[(4-Amino-6-N-substituted-1,3,5-triazin-2-yl)methylthio]-4-chloro-5-methyl-N-(1H-benzo[d]imidazol-2(3H)-ylidene)Benzenesulfonamides
Source: Int J Mol Sci. 2020 Apr 22;21(8):2924. doi: 10.3390/ijms21082924 (PMC7215599; doi:10.3390/ijms21082924)
Supplement: Supplementary file 1 [file ijms-21-02924-s001.pdf]

Supplementary materials

Synthesis, Antitumor Evaluation, Molecular Modeling and Quantitative Structure–Activity Relationship (QSAR) of Novel 2-[(4-Amino-6-*N*-substituted-1,3,5-triazin-2-yl)methyl thio]-4-chloro-5-methyl-*N*-(1*H*-benzo[*d*]imidazol-2(3*H*)-ylidene)Benzenesulfonamides

Łukasz Tomorowicz [1], Jarosław Sławiński [1,*], Beata Żołnowska [1,*], Krzysztof Szafrański [1] and Anna Kawiak [2]

[1] Department of Organic Chemistry, Medical University of Gdańsk, Al. Gen. J. Hallera 107, 80-416 Gdańsk, Poland; ltomorowicz@gumed.edu.pl (Ł.T.); k.szafranski@gumed.edu.pl (K.S.)

[2] Department of Biotechnology, Intercollegiate Faculty of Biotechnology, University of Gdańsk and Medical University of Gdańsk, ul. Abrahama 58, 80-307 Gdańsk, Poland; anna.kawiak@biotech.ug.edu.pl

* Correspondence: jaroslaw@gumed.edu.pl (J.S.); zolnowska@gumed.edu.pl (B.Ż.); Tel.: +48 58 349 10 98 (J.S.); +48 58 349 12 29 (B.Z.)

Spectrum 1. ^{1}H NMR of compd **6 AGS-655** (500 MHz, DMSO-*d*$_6$).

Spectrum 2. ^{13}C NMR of compd **6 AGS-655** (125 MHz, DMSO-*d*$_6$).

Spectrum 3. ^{1}H NMR of compd **9 AGS-649** (500 MHz, DMSO-*d*$_6$).

Spectrum 4. ^{13}C NMR of compd **9 AGS-649** (125 MHz, DMSO-*d*$_6$).

Spectrum 5. ^{1}H NMR of compd **11 AGS-659** (500 MHz, DMSO-*d*$_6$).

Spectrum 6. ^{1}H NMR of compd **19 AGS-651** (500 MHz, DMSO-*d*$_6$).

Spectrum 7. ^{13}C NMR of compd **19 AGS-651** (125 MHz, DMSO-*d*$_6$).

Spectrum 8. ^{1}H NMR of compd **31 AGS-676**(500 MHz, DMSO-*d*$_6$).

Spectrum 9. ^{13}C NMR of compd **31 AGS-676** (125 MHz, DMSO-*d*$_6$).

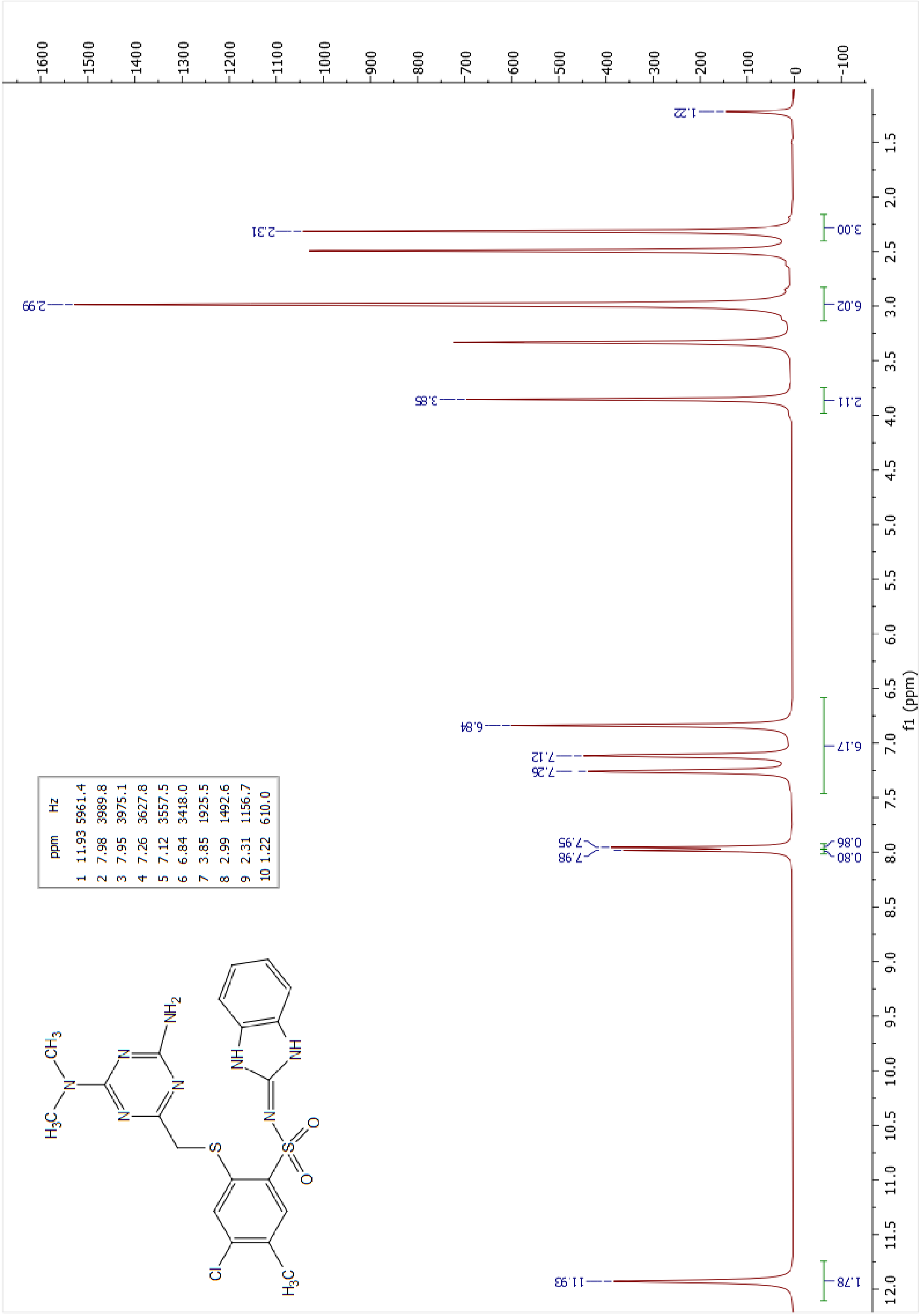

Spectrum 1. ^{1}H NMR of compd **6** (**AGS-655)** (500 MHz, DMSO-d_6)

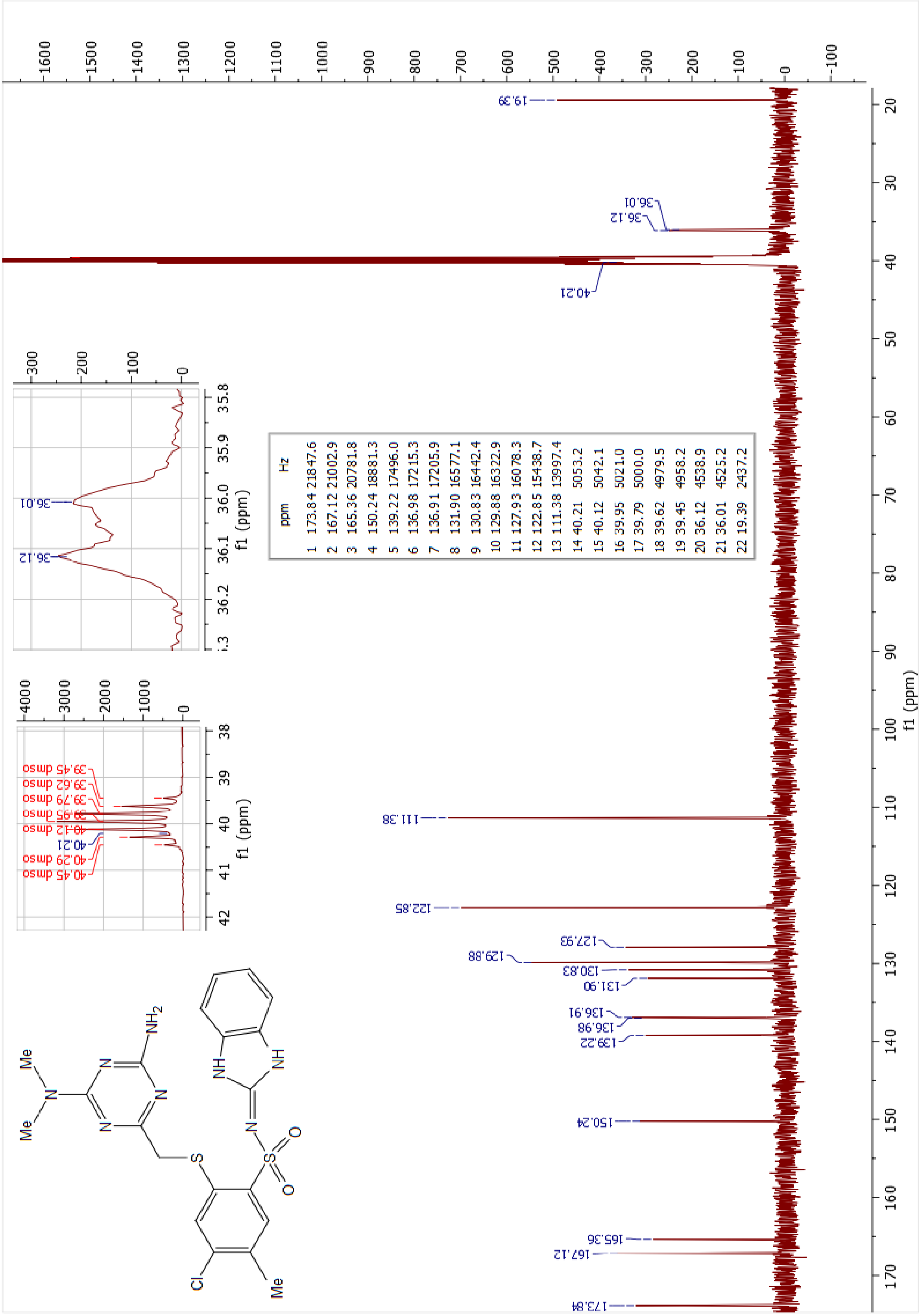

Spectrum 2. ^{13}C NMR of compd **6 AGS-655** (125 MHz, DMSO-d_6)

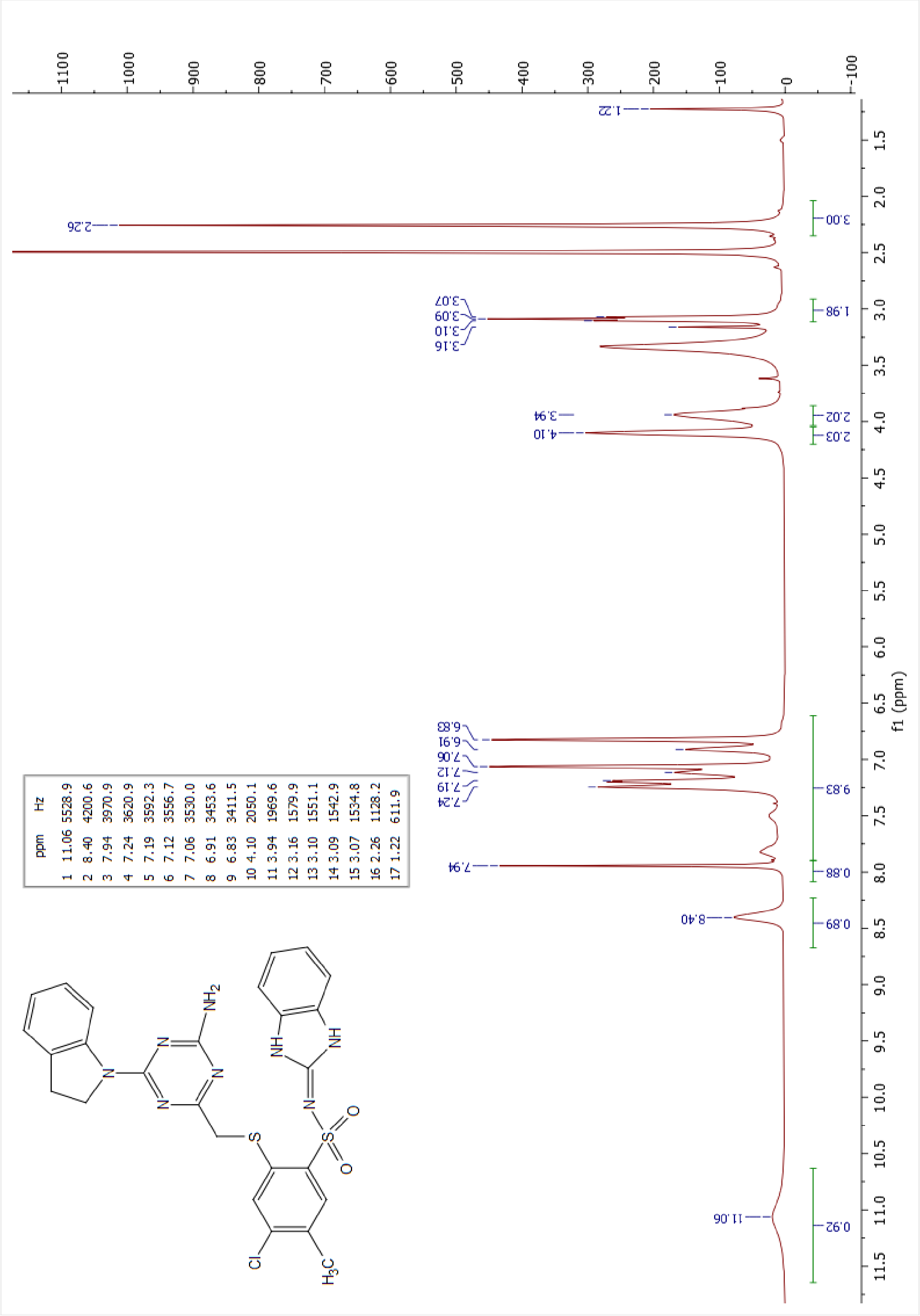

Spectrum 3. ^{1}H NMR of compd **9 AGS-649** (500 MHz, DMSO-d_6)

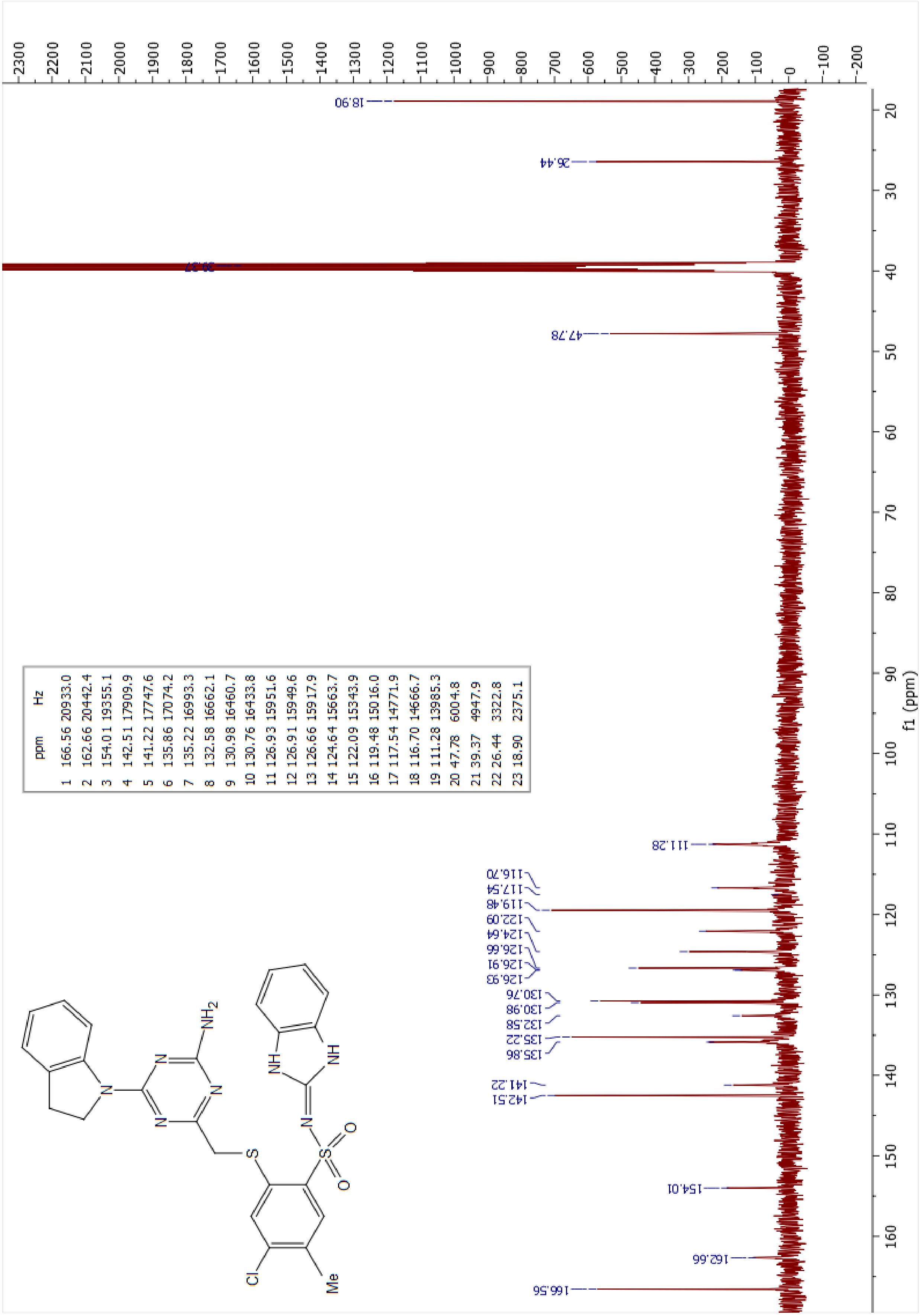

Spectrum 4. ^{13}C NMR of compd **9 AGS-649** (125 MHz, DMSO-d_6)

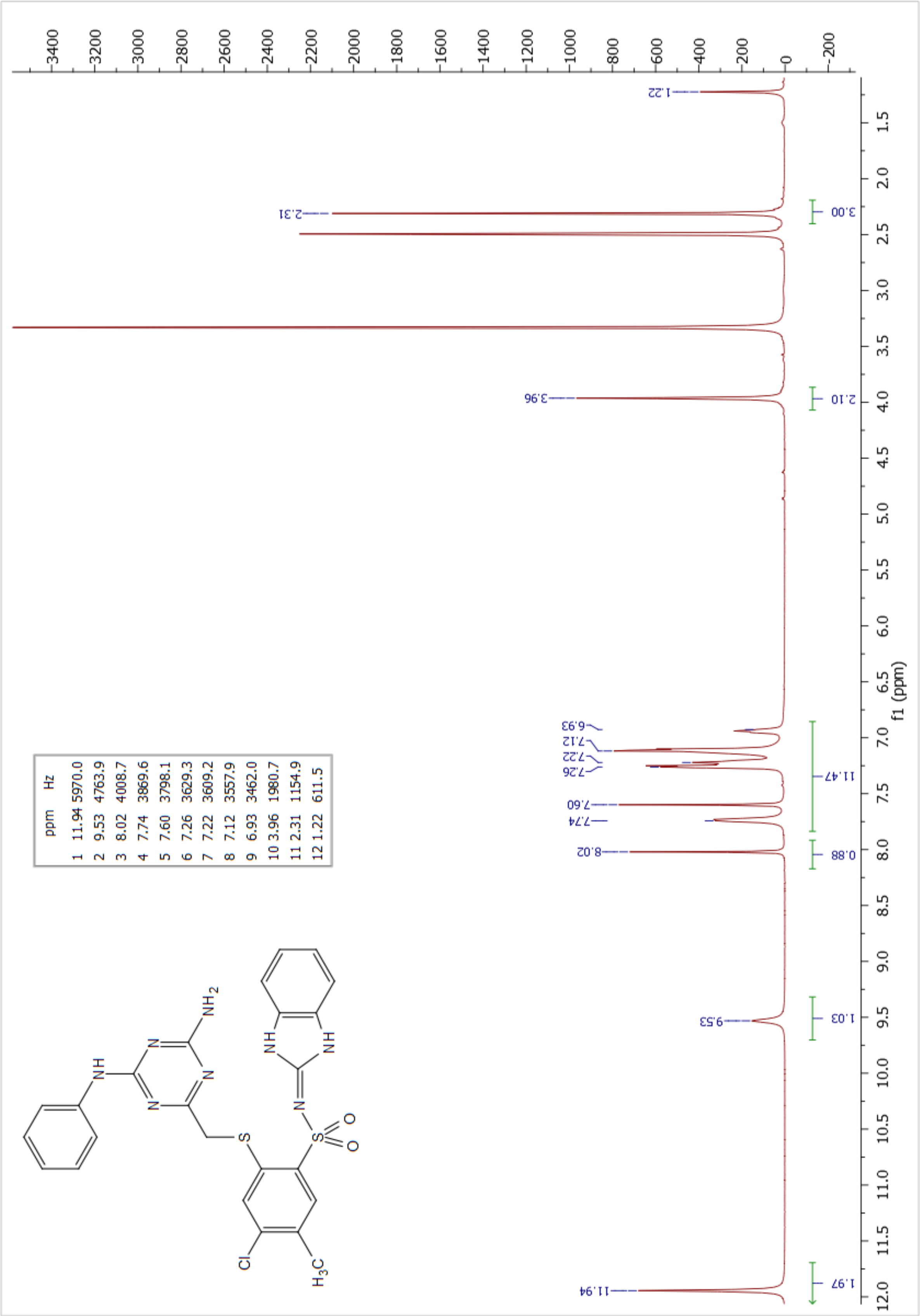

Spectrum 5. ^{1}H NMR of compd **11** (**AGS-659)** (500 MHz, DMSO-*d*₆)

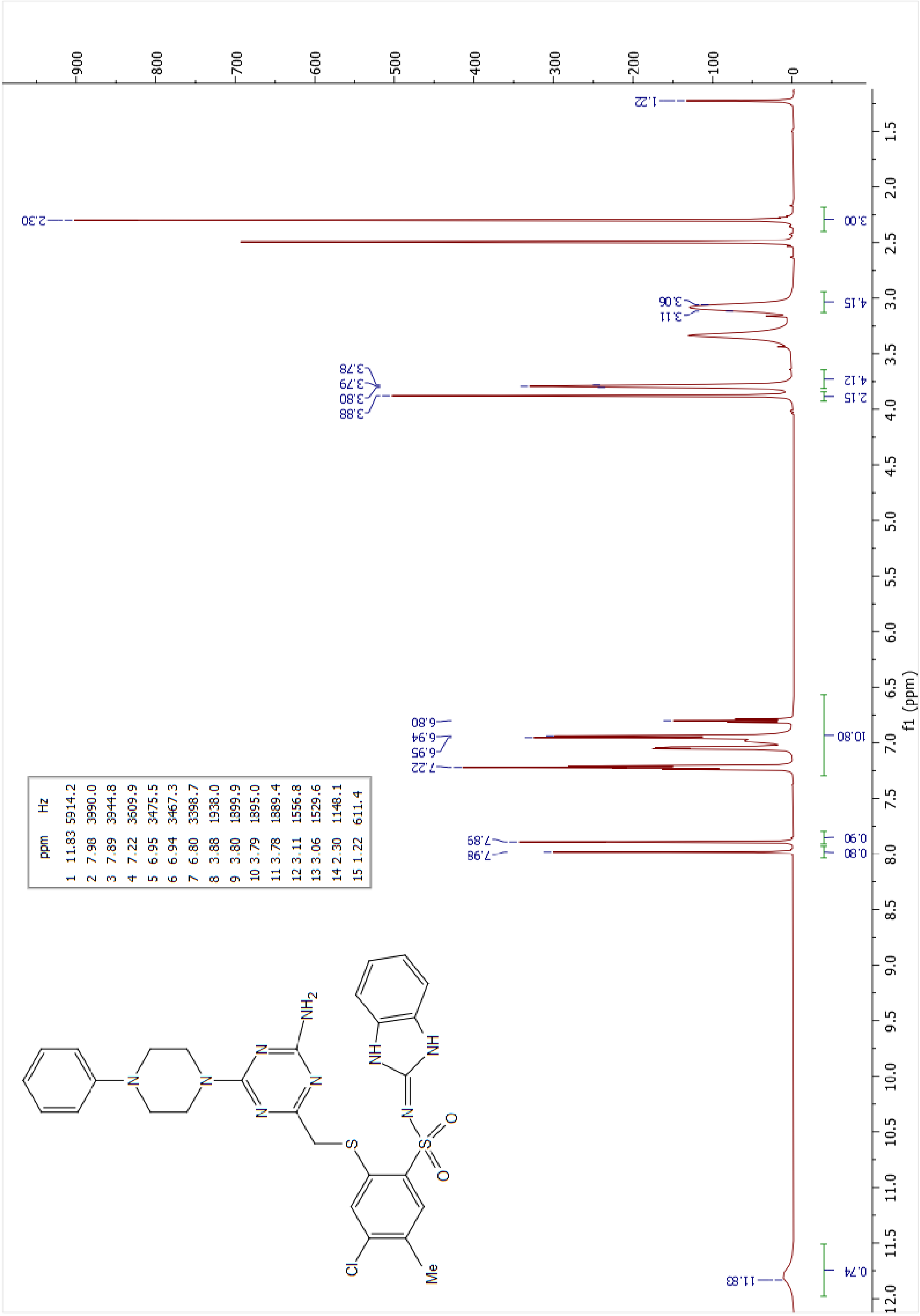

Spectrum 6. ^{1}H NMR of compd **19 AGS-651** (500 MHz, DMSO-d_6).

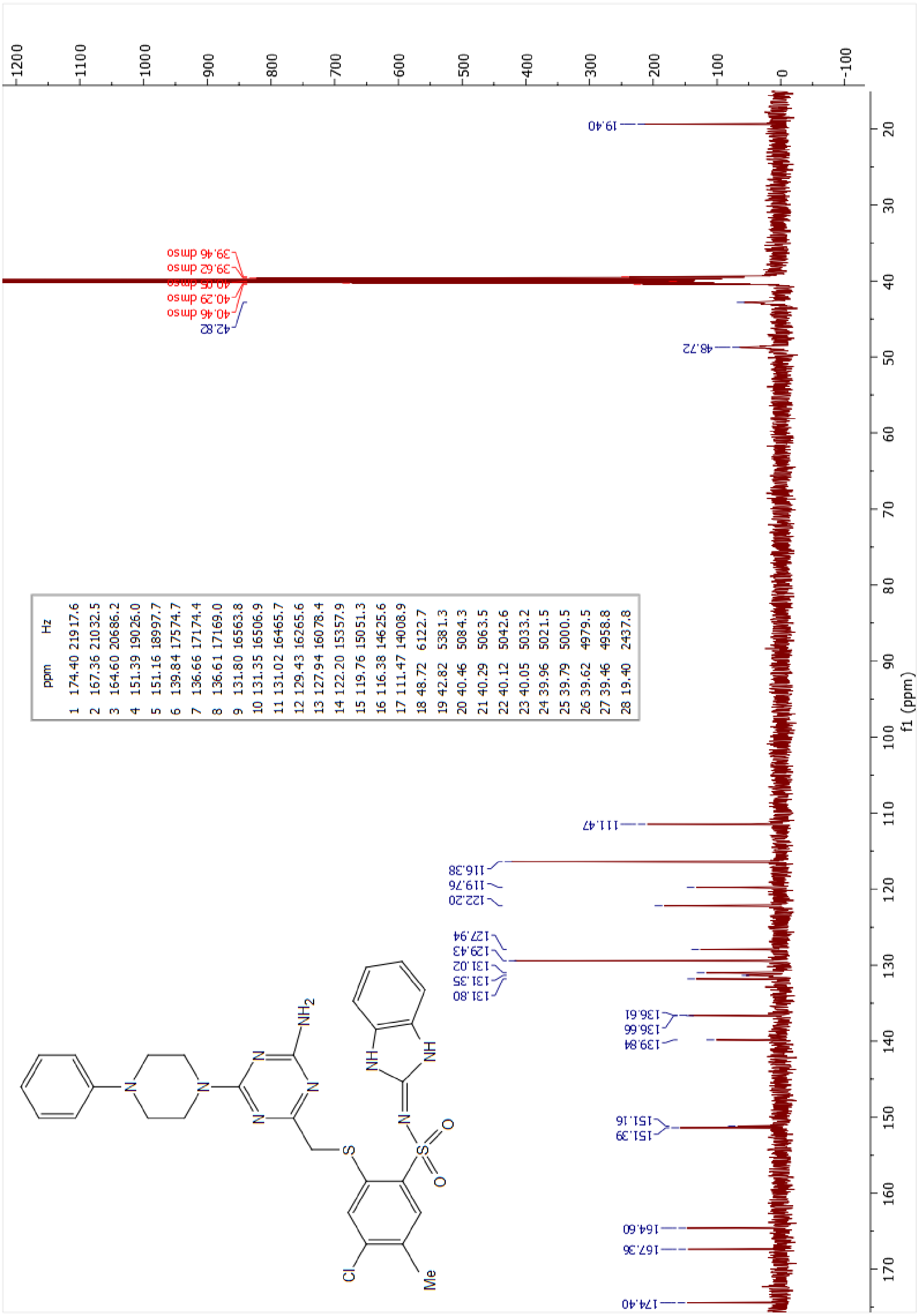

Spectrum 7. ^{13}C NMR of compd **19 AGS-651** (125 MHz, DMSO-d_6)

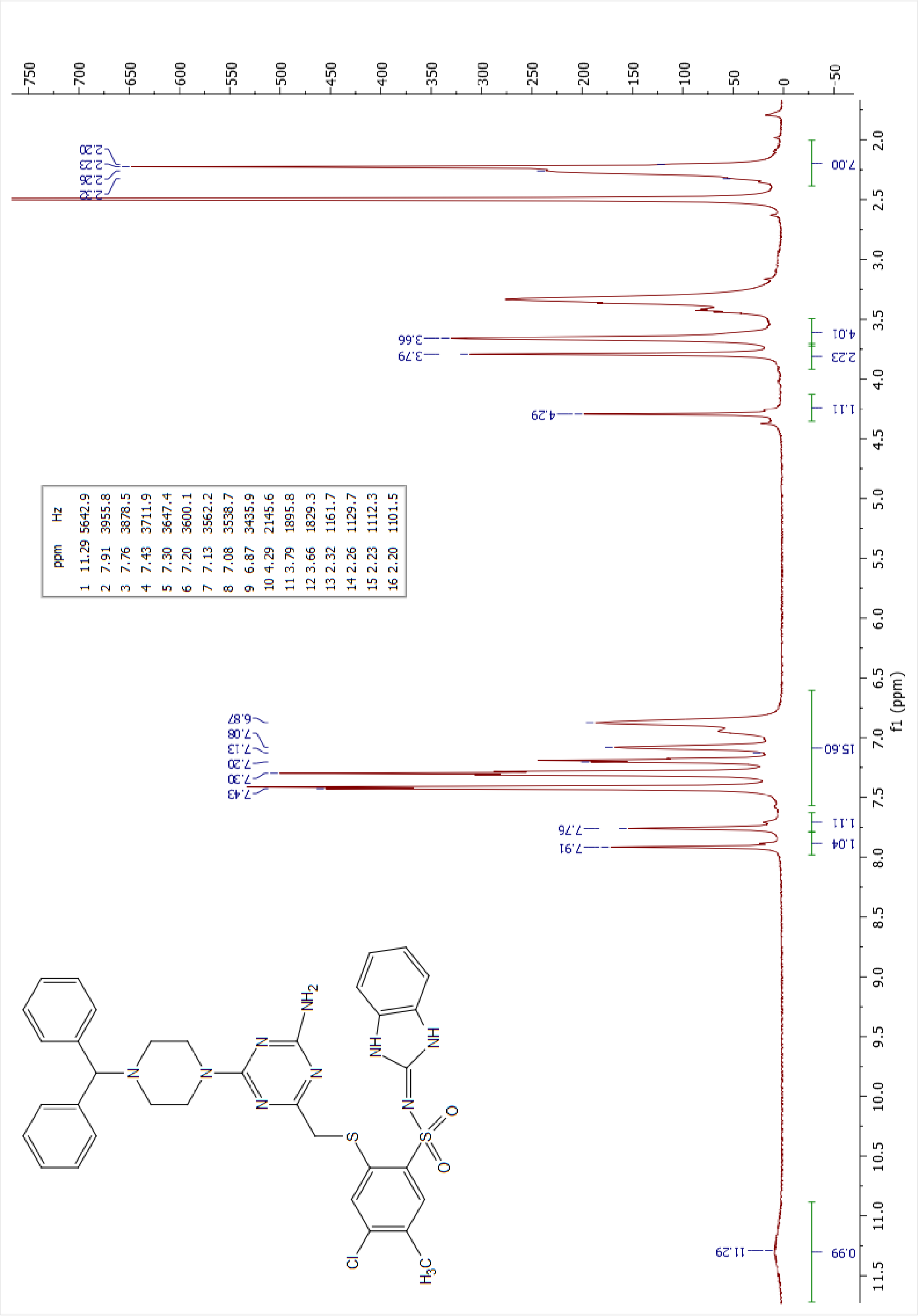

Spectrum 8. ^{1}H NMR of compd **31 AGS-676**(500 MHz, DMSO-d_6)

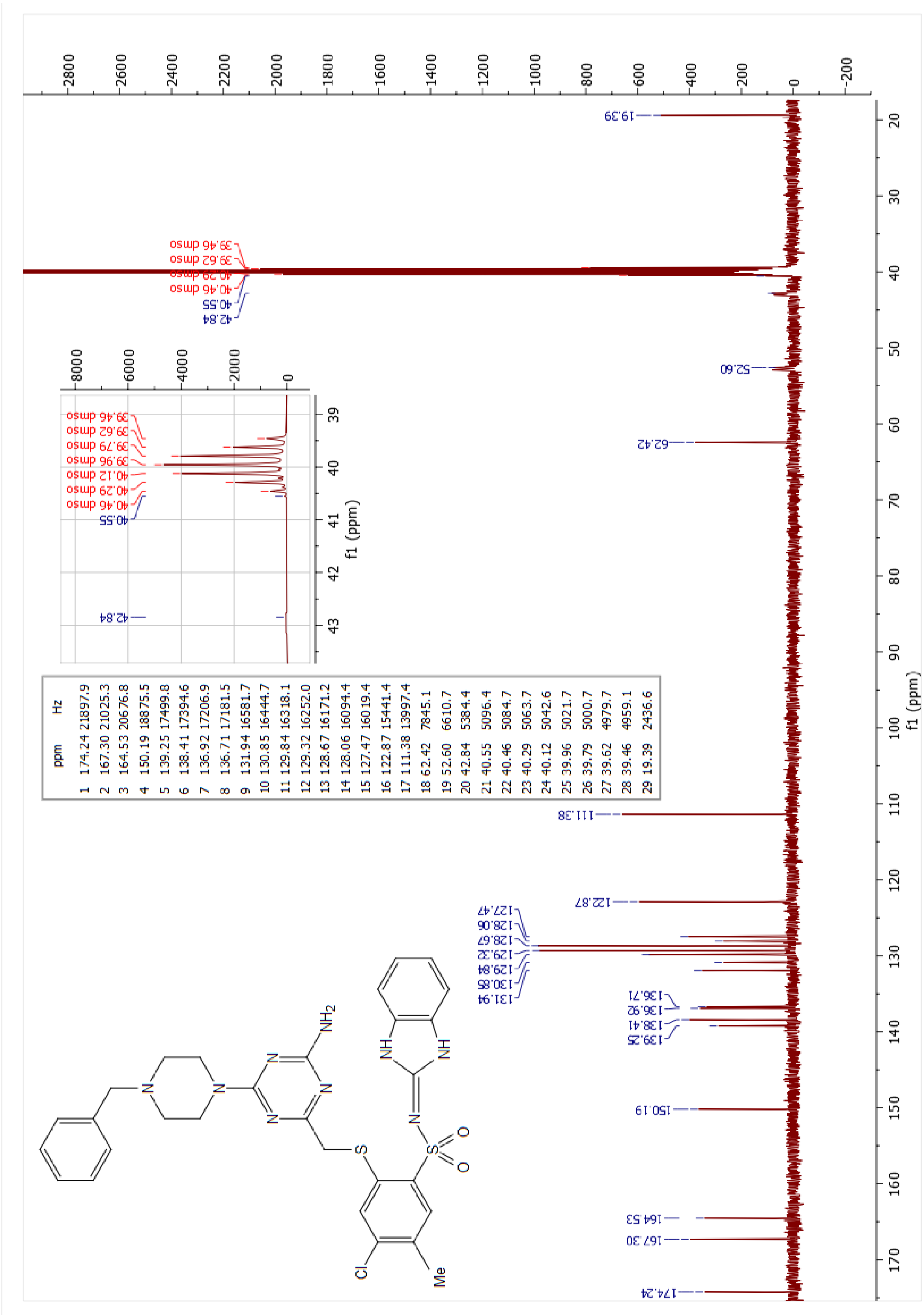

Spectrum 9. ^{13}C NMR of compd **31 AGS-676** (125 MHz, DMSO-d_6)
